# Respiratory Illness in Households of School-Dismissed Students during Influenza Pandemic, 2009

**DOI:** 10.3201/eid1709.101589

**Published:** 2011-09

**Authors:** Nicole J. Cohen, David B. Callahan, Vanessa Gonzalez, Victor Balaban, Rose T. Wang, Paran Pordell, Ricardo Beato, Otilio Oyervides, Wan-Ting Huang, Mehran S. Massoudi

**Affiliations:** Author affiliations: Centers for Disease Control and Prevention, Atlanta, GA, USA

**Keywords:** influenza, viruses, pandemic, schools, prevention and control, disease outbreaks, respiratory illness, households, students, respiratory illness, school closure, Chicago, United States, letter

**To the Editor:** In response to the emergence of pandemic (H1N1) 2009 virus (*1*), the Centers for Disease Control and Prevention (CDC) issued interim guidance for preventing spread of the pandemic virus in schools. Initial guidance recommended that dismissal of students be considered for schools with confirmed cases of pandemic (H1N1) 2009 infection. The guidance was subsequently revised to recommend monitoring for respiratory illness and exclusion of ill students until they were noninfectious, rather than dismissal.

In Chicago, Illinois, USA, the first cases of pandemic (H1N1) 2009 infection were identified on April 28, 2009, of which 1 occurred in an elementary school student (*2*). In accordance with CDC guidance at the time, the school (school A) was closed for 1 week, April 29–May 5, 2009. CDC and the Chicago Department of Public Health investigated respiratory illnesses among students and their households during the period surrounding the school closure.

A telephone survey of students’ households was conducted during May 15–20, 2009 (*3*). One adult member of each household was asked whether any household members had been “sick with cold or flu symptoms or fever” since April 12. Age, date of illness onset, and symptoms and signs (fever, cough, sore throat, rhinorrhea or nasal congestion [runny or stuffy nose]) were recorded. Acute respiratory illness was defined as >1 symptom or sign from the list provided. Influenza-like illness was defined as fever plus cough or sore throat. Reports were excluded if onset date was before April 12 or unknown. Descriptive analysis was performed, and household attack rates were calculated. Dates of onset were used to evaluate timing of illness in relation to school closure and possible transmission within households. The investigation was approved as nonresearch by CDC.

Of 609 eligible households, 439 (72%) had a working telephone number, of which 170 (39%) completed the survey. Thirty-nine (23%) households, representing 181 persons, reported 58 illnesses that met the acute respiratory illness definition, of which 37 (64%) also met the influenza-like illness definition. Median age was 10 years (range <1–48 years). Of 57 household members for whom age and student status were recorded, 42 (74%) were students at school A. Thirty-four (60%) reported onset of symptoms before or on the day of school dismissal (Figure).

**Figure:**
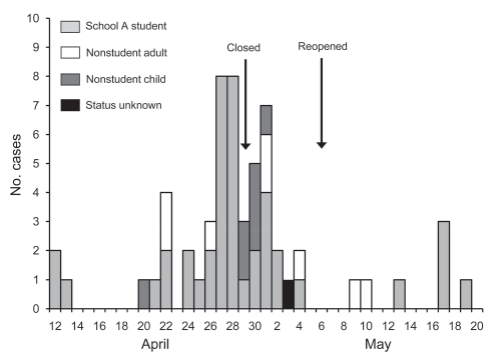
Respiratory illness in households of school-dismissed students during the pandemic (H1N1) 2009 outbreak, Chicago, Illinois, USA, 2009. Arrows indicate dates when school A closed and reopened.

Household attack rates ranged from 10% to 100% (median 25%). Five (13%) households reporting illness had no ill students who attended school A. In 4 of 11 households reporting >2 illnesses, students became ill before nonstudent household members. In the remaining 7 households, onset dates did not suggest student-to-nonstudent transmission.

Even though the school was closed almost immediately after the first pandemic (H1N1) 2009 case was confirmed in a student, onset of ≈60% of reported illnesses occurred before or on the day of school dismissal, suggesting that unrecognized transmission was already occurring in the school or community. These results are supported by data on confirmed cases of pandemic (H1N1) 2009 in Chicago, which suggest that community transmission was high during the survey period (*2*). Our results also indicated that at least some illness among school A households originated from sources other than the school and support the approach of considering school dismissal only in conjunction with other community mitigation strategies.

In Hong Kong Special Administrative Region, People’s Republic of China, where all primary schools, kindergartens, and child care centers were immediately closed for 14 days after identification of the first local case of pandemic (H1N1) 2009, school closures were concluded to have substantially decreased transmission (*4*). The applicability of these findings to communities where such sweeping measures might be less acceptable is unclear.

If school dismissal is considered as a strategy, dismissal early in the pandemic most likely would have the most impact, depending on duration of dismissal, other mitigation measures, and compliance with social distancing recommendations (which was mixed during the 2009 pandemic [3,5]). Polling of parents whose children experienced school dismissal showed high acceptance of short-term (3–5 days) dismissals and low economic impact, especially on lower income families (*3**,**6*). However, dismissal for longer periods needs to be balanced by the adverse impact on education, loss of student services, and socioeconomic impact on families (*7**–**9*).

This investigation was limited by the relatively low response rate; however, demographics for the sample in our study were similar to those of the school as a whole (*3*). Other limitations included the exclusive use of reported symptoms to document illness, possible unrecognized asymptomatic cases, and absence of similar data from later in the pandemic. The 1-week closure period might not have provided enough information to capture any effect, and comparative data were not available from schools that were not dismissed during the pandemic. Further investigation is needed to evaluate the efficacy and impact of school dismissal, including the timing of dismissal in relation to recognition of cases in a school or community and the impact of school dismissal relative to other community mitigation strategies.
